# The value relevance of digital marketing capabilities to firm performance

**DOI:** 10.1007/s11747-022-00858-7

**Published:** 2022-04-20

**Authors:** Christian Homburg, Dominik M. Wielgos

**Affiliations:** 1 University of Mannheim, 68131 Mannheim, Germany; 2 Alliance Manchester Business School, Manchester, M13 9SS UK

**Keywords:** Digital marketing, Digital marketing capabilities, Marketing strategy, Firm performance

## Abstract

Digital transformation of the marketing organization forces firms to develop new digital marketing capabilities (DMCs) to remain competitive. However, despite considerable academic and managerial interest, the value relevance of DMCs beyond the value achieved through classic marketing capabilities (CMCs) remains unclear. Similarly, research investigating the interaction effect of DMCs and CMCs is scarce. We address both research gaps by drawing on a mixed-methods approach combining in-depth interviews and a multi-industry, multisource dataset. The results reveal that DMCs significantly contribute to firm profitability beyond the influence of CMCs. Drawing on the contingent view of resource-based theory, we investigate the moderating influence of organizational and environmental contingencies on the interaction effect of DMCs and CMCs. This investigation reveals important tradeoffs that result in actionable managerial implications for realizing the complementarity potential—and preventing the substitutive potential—of a firm’s DMCs and CMCs.

**Supplementary Information:**

The online version contains supplementary material available at 10.1007/s11747-022-00858-7.

Marketing capabilities are complex bundles of firm-level skills and knowledge embedded in organizational processes that carry out marketing tasks and firm adaptation to marketplace changes (Moorman & Day, 2016; Morgan et al., 2012, 2018). They are widely accepted as significant drivers of firm performance (Moorman & Day, 2016). However, to keep up with “profound transformations in practice enabled by the digitization of marketing activities” (Moorman & Day, 2016, p. 6), firms are forced to develop “[n]ew digital capabilities and, specifically, digital marketing capabilities” (Verhoef & Bijmolt, 2019, p. 4).

*Digital marketing capabilities* (DMCs) refer to a firm’s ability to use digital technology–enabled processes to interact with customers and partners in a targeted, measurable, and integrated way to create new forms of value without regard for distance or time (Kannan & Li, 2017; McIntyre & Virzi, 2019; Sridhar & Fang, 2019). Although managers’ interest in digital marketing has generated a rich body of literature that has substantially contributed to the marketing discipline (see Kannan & Li, 2017; Lamberton & Stephen, 2016; Yadav & Pavlou, 2014), research in the domain of DMCs is scarce.^1^ This scarcity is particularly surprising, as both academics (Kannan & Li, 2017; Moorman, 2016; Verhoef & Bijmolt, 2019) and practitioners (Galante et al., 2013; Sayre et al., 2012) have repeatedly “urge[d] scholars to examine these newer capabilities” (Moorman & Day, 2016, p. 12). Despite their valuable contributions, the few empirical studies that have investigated capabilities in digital marketing are subject to at least two major limitations.

First, no clear understanding exists of whether DMCs matter to firm performance (see Table 1). This is because empirical research investigating the contribution of DMCs to firm performance is scant. More specifically, most studies tend to narrowly examine firm capabilities related to single digital marketing activities (e.g., Nguyen et al., 2015; Trainor et al., 2014; Wang & Kim, 2017). Consequently, empirical evidence on whether developing firm capabilities across a broad set of digital marketing activities (e.g., social media marketing, mobile marketing, content marketing) really pays off is limited. More important, prior studies have not accounted for the importance of DMCs relative to *classic marketing capabilities* (CMCs) for deploying marketing-mix-related processes (see Table 1). Thus, the value relevance of DMCs beyond the effects of CMCs remains unclear (Palmatier, 2016).

**Table 1:**
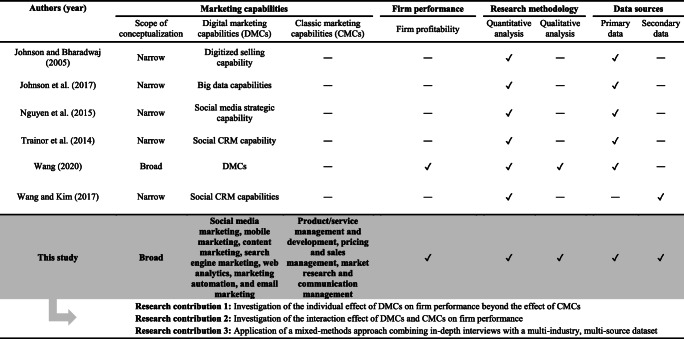
Relevant empirical research on digital marketing capabilities

Notes: **✓** included in the study; **―** not included in the study.

Recent reports from business practice underscore the managerial relevance of this research inquiry. For example, results from IBM’s Global C-Suite Study conducted among more than 2000 chief marketing officers (CMOs) reveal that organizations “have been challenged to grow their skillsets beyond core marketing capabilities for some time now” (IBM Institute for Business Value, 2018, p. 7) to remain competitive in the digital age. Consequently, managers are facing the tension of allocating their scarce resources between building new DMCs and relying on established CMCs. A marketing and sales director from the automotive industry, whom we interviewed for this study, emphasized this challenge:

You have to move away from classic marketing, 4 Ps or however you call it. Digital transformation means above all digital [marketing] capabilities.… The problem you have, or everyone has is that there is no extra marketing budget. Everyone has probably told you that. That means that the art now is to cut off a part from the classic marketing activities and make the first attempts in digital. [However], this usually goes completely wrong at first.

Recent results from the CMO Survey (2019) further underscore this managerial challenge. Although firms across sectors have assigned top priority to increasing their overall marketing capability investments, marketing executives report that the contribution of DMCs to firm performance falls far behind expectations. As demonstrating the financial accountability of marketing investments remains one of the key challenges for CMOs, this shortfall may be one of the reasons most marketing leaders still tend to focus on “managing the present” rather than “preparing for the future” (The CMO Survey, 2019). Yet an overreliance on classic core capabilities carries the danger of losing competitive advantage when firms face profound technological changes (Leonard-Barton, 1992). Taken together, a pressing managerial concern is to empirically substantiate the relevance of DMCs (vs. CMCs) “to firm performance to assess the key-capabilities that are driving … success” (Verhoef & Bijmolt, 2019, p. 346).

A second major research gap is the interaction effect of DMCs and CMCs. This research inquiry is managerially relevant because (1) capabilities coexist in firms and are often intertwined (Feng et al., 2017) and (2) many firms struggle to exploit the complementarity potential of their DMCs and CMCs (Sridhar & Fang, 2019; The CMO Survey, 2018). Lack of complementarity may be one important reason CMOs cannot achieve the expected performance benefits from their substantial marketing technology investments, which account for nearly 30% of their marketing budgets (McIntyre & Virzi, 2019; The CMO Survey, 2018). Anecdotal evidence cites inappropriate organizational conditions as key impediment to take full advantage of a firm’s DMCs and CMCs (Armstrong et al., 2020). This is apparent when considering empirical results from a different but related field of research: Studies in the advertising context report non-significant (e.g., Bayer et al., 2020), positive (e.g., Kumar et al., 2017), or negative (e.g., Sridhar et al., 2016) interaction effects of online and offline advertising on firm performance. Such inconsistent results imply the existence of contingencies that may either enable or prevent firms from realizing the full potential of their digital and classic marketing practices. In the marketing capability context, however, insights are scarce on whether and when DMCs and CMCs function as complements or substitutes.

Against this background, this study takes an initial step toward closing these research gaps and makes three key contributions (see Table 1). First, this study adopts a more holistic approach and simultaneously investigates the performance effects of DMCs and CMCs, each conceptualized across a broad set of marketing activities. The results reveal that DMCs contribute significantly to firm profitability.

Second, we provide a deeper understanding of the interaction effect of DMCs and CMCs. Following Wade and Hulland (2004), we suggest that organizational contingencies and environmental contingencies either enable a firm to realize the complementarity potential of its capabilities or prevent it from doing so. Regarding organizational contingencies, the results show that a customer orientation helps realize the complementarity potential of DMCs and CMCs over and above the contribution of each capability type, thereby resulting in higher levels of firm profitability. By contrast, a competitor orientation hampers the interaction effect of DMCs and CMCs, thereby having a negative impact on firm profitability. Regarding environmental contingencies, our study reveals that environmental dynamism positively moderates the interaction between DMCs and CMCs. Thus, we reveal important tradeoffs that result in actionable managerial implications for realizing the complementarity potential—and preventing the substitutive potential—of a firm’s marketing capabilities.

Third, we draw on a mixed-methods approach combining in-depth interviews and a unique multi-industry, multisource dataset, which allows us to enhance the validity and generalizability of the study results (Davis et al., 2011). We draw on primary data to capture DMCs and CMCs because knowledgeable key informants “provide the most direct measures of [marketing] capabilities” (Danneels, 2016, p. 2175). Given that valid secondary data are generally not available to measure DMCs and CMCs across a broad set of marketing capabilities and firms from various industries, primary data are essential to test our hypotheses. Importantly, we use secondary data to measure firm profitability. According to Morgan et al.’s (2019) systematic review of marketing strategy research, such a research design is rare. In addition, it allows us to overcome the methodological limitations of prior studies that relied on either single-source primary data or secondary data. Thus, our study is the first to combine primary and secondary data and test the *individual* and *interactive* effects of DMCs and CMCs on firm performance (see Table 1).

## Development of conceptual framework

**Fig. 1:**
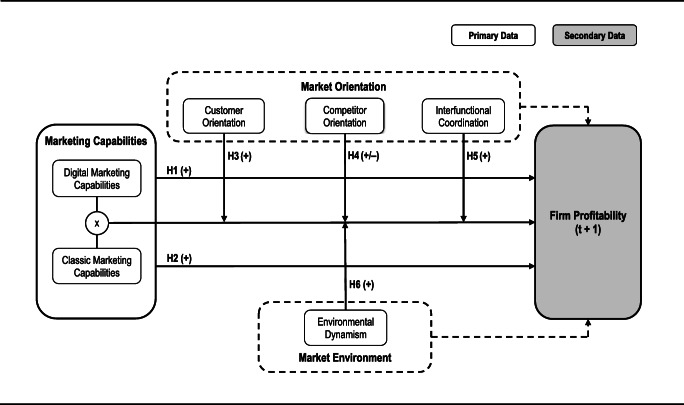
Conceptual framework

In this section, we develop our conceptual framework (Fig. 1), which is rooted in resource-based theory (RBT), to investigate the value relevance of DMCs beyond the value achieved through CMCs and related contingencies. Following prior research (e.g., Sarin & Mahajan, 2001; Schmitz et al., 2014), we applied a two-step procedure. In the first step, we conducted in-depth interviews to gain a deeper understanding of our focal construct. We interviewed 49 managers—including eight C-level executives—from various industries, hierarchical levels, and functions who were key decision makers in the planning, design, and execution of their firms’ customer-facing digital initiatives. Web Appendix A details the sample characteristics and the qualitative research design. In the second step, we enriched theoretical insights from RBT with managerial insights from our interviews to enhance the external validity of our conceptual framework, ensure the meaningfulness of the selected constructs, and inform our quantitative research design (Davis & Golicic, 2010; Deshpandé, 1983).

### Conceptualization of marketing capabilities

In our conceptual framework, we distinguish between DMCs and CMCs. Drawing on literature-based and interview-based insights, we define DMCs as a firm’s ability to use digital technology–enabled^2^ processes to interact with customers and partners in a targeted, measurable, and integrated way to create new forms of value without regard for distance or time (Kannan & Li, 2017; McIntyre & Virzi, 2019; Sridhar & Fang, 2019). CMCs refer to a firm’s ability to use marketing-mix-related processes of product/service management and development, pricing and sales management, and market research and communication management to achieve desired marketing outcomes (Morgan 2012; Morgan et al., 2018). As Table 2 shows, DMCs and CMCs differ in terms of (1) scalability, (2) measurability, (3) interconnectivity, and (4) adaptability.

**Table 2:** Conceptual differences between digital and classic marketing capabilities

Characteristics	Digital marketing capabilities	Classic marketing capabilities	Literature support
Scalability	High	Low	e.g., Day (2011), Kannan and Li (2017), Li et al. (2021), Sridhar and Fang (2019), Wedel and Kannan (2016)
Measurability	High	Moderate	
Interconnectivity	High	Low	
Adaptability	High	Moderate	

Notes: This table displays average values. For example, the degree of scalability of digital marketing capabilities is high on average, while the degree of scalability of classic marketing capabilities is low on average

First, DMCs are characterized by a higher degree of scalability than CMCs. Scalability is the degree to which a firm is able to generate increasing returns in the marketplace with an under proportionate input of additional resources (Nielsen & Lund, 2018; Zhang et al., 2015). Although CMCs have high revenue potential, they incur significantly higher deployment costs than DMCs (Sridhar & Fang, 2019). This view is confirmed by Goldfarb and Tucker (2019, p. 281), who note that compared with classic marketing, “[d]igital marketing is inherently different … due to a reduction of five categories of costs: Search, reproduction, transportation, tracking, and verification.” DMCs foster the implementation of highly scalable content-based (i.e., selling digital content), information-based (i.e., selling customer information), or advertising-based (i.e., selling the customers’ attention in the form of online advertising) revenue models and combinations thereof. In this way, DMCs have the potential to generate higher revenues at comparably lower costs than CMCs through the provision of digital goods that are non-rival and have low or even zero deployment costs (Kannan & Li, 2017; Lambrecht et al., 2014). Regarding the high scalability potential of DMCs, the marketing manager of a B2B internet firm emphasized the role of digital content:

In general, content is the most cost-effective marketing channel, simply because once you’ve got a piece of content, which performs well, you can repurpose it with little effort, or just leave it as is and it will continue to generate increasing traffic, leads, email subscribers and so on, just because it is out there. Since the content is providing value, more and more customers will engage with your firm and eventually become paying customers. This is particularly what we observe with SaaS companies in our industry, which experience high growth.

Second, DMCs are characterized by a higher degree of measurability than CMCs. Measurability is the degree to which a firm is able to generate and assess information related to its activities in the marketplace (Homburg et al., 2012a; O'Sullivan & Abela, 2007). Increasing pressure on marketing to demonstrate its value to the firm has led to the development of sophisticated marketing performance measurement systems that help firms measure the efficiency and effectiveness of CMCs (O'Sullivan & Abela, 2007). Nevertheless, the measurability of CMCs is generally lower than that of DMCs. This is because DMCs are inherently data-driven, thus providing managers with higher volume, variety, and velocity of data than CMCs (Johnson et al., 2017; Li et al., 2021). While this imposes new challenges for marketing analytics, it still enables companies to generate more information about and better evaluate the performance of marketing activities (Wedel & Kannan, 2016). Overall, DMCs enable more precise targeting of individual customers and more accurate attribution of causes and effects of marketing activities to individual customers, which is particularly evident in the advertising context (Bayer et al., 2020). As a group brand manager from the consumer goods industry noted:

Digital marketing is more data-driven than traditional marketing. For example, social media gives you the opportunity to analyze the performance of every single post, how many likes has it achieved, how good is it in terms of range, how is the interaction with our customers, and so on. In the past, we had a TV spot, we did survey-based market research, or invited customers to our studio to gather market feedback. In the social media area, you get feedback from customers in real-time, learn from it, and improve your marketing activities.

Third, DMCs are characterized by a higher degree of interconnectivity than CMCs. Interconnectivity is the degree to which a firm is able to establish and leverage interactive linkages with existing and new customers and partners in the marketplace (Bellamy et al., 2014; Liu et al., 2020). A “key focus for DMCs is to enhance linkages with customers, suppliers and channel partners” (Wang, 2020, p. 559), thus providing new sources of value creation through novel interaction possibilities and access to new markets within and outside a firm’s current industry boundaries. In contrast with CMCs (e.g., customer relationship management [CRM]), which primarily rely on firm-initiated interactions, DMCs (e.g., social media) enable new ways of firm- and customer-initiated interactions (Li et al., 2021; Malthouse et al., 2013). This empowers both—firms and customers—to better leverage their relationships by more actively listening to and engaging with each other. For example, the chief digital officer of a home appliances manufacturer illustrated the importance of mobile marketing for gaining unique access to customers, which was heretofore impossible:

[In the old world], we sell the product to the retailer and the retailer sells it to a customer. We don’t know our customers in the old world, we don’t really need to focus on interacting with them, we don’t have any opportunities to do something good for them…. In the digital world, we have a product that can be connected via a mobile application and where I as a manufacturer see exactly what the customer does with it. In other words, I know much more than the retailer, much more than any market research, and much more than product development. I am suddenly able to personally interact with the customer and find out how to do something good for him.

Fourth, DMCs are characterized by a higher degree of adaptability than CMCs. Adaptability is the degree to which a firm is able to rapidly sense and respond to changes in the marketplace (Day, 2011; Guo et al., 2018). While CMCs certainly allow firms to sense and respond to market opportunities and threats, the time span between market change and firm response is typically shorter for DMCs. For example, compared with A/B testing, which enables firms to adapt mobile apps or online advertising in real time, conducting survey-based market research and then adapting product features or offline advertising campaigns are typically very time-consuming (Guo et al., 2018). Day (2011, p. 183) recognizes the limited adaptability of CMCs by alluding to the need to develop DMCs that “are adaptive and enable the firm to adjust its strategies to fit fast-changing markets.” For example, the COVID-19 pandemic has shown that DMCs are critical to adapt to large-scale social distancing and continue the relationship with customers (The CMO Survey, 2021). As a regional marketing manager from a consumer goods firm noted:

The main difference that I see is in the speed… Digital marketing is much faster and much more adaptable than traditional marketing. In traditional marketing if you were doing market research, all the research and analysis took a long time, whereas in digital marketing you can generate and act on market insights overnight.

### Marketing capabilities and firm performance

RBT relies on two key assumptions (Kozlenkova et al., 2014). First, the resource heterogeneity assumption posits that some firms are more skilled than others because they possess unique bundles of resources, defined as stocks of a firm’s tangible and intangible assets (Amit & Schoemaker, 1993; Morgan 2012). Second, the resource immobility assumption argues that, owing to the difficulty of obtaining and trading resources, firms vary in the ability to acquire and deploy resources, ultimately resulting in performance differences (Amit & Schoemaker, 1993; Barney, 1991). According to RBT, a firm’s capabilities are among the most critical drivers of firm performance (Kozlenkova et al., 2014).

Following prior marketing capability research (Feng et al., 2017; Morgan et al., 2009a, b), we aim to examine the direct effects of DMCs and CMCs on firm profitability (Fig. 1). Specifically, we consider return on assets (ROA), which indicates how effective a firm is in deploying its resources to generate profits (Katsikeas et al., 2016). As marketing capabilities enable firms to achieve competitive advantage through the effective transformation of resource inputs into outputs (Kozlenkova et al., 2014), ROA is of clear theoretical relevance for our study from an RBT perspective. Indeed, ROA is a dominant measure of firm performance in marketing capability research (e.g., Feng et al., 2015; Leonidou et al., 2013; Morgan et al., 2009b; Vorhies et al., 2011; Vorhies & Morgan, 2005).

In addition to positing the individual value relevance of different capabilities to firm performance, RBT argues that capabilities can interact with one another (Kozlenkova et al., 2014). Capabilities are *complementary* when a positive interaction exists or the marginal benefit of one capability increases as the level of the other capability increases. By contrast, capabilities are *substitutive* when a negative interaction exists or the marginal benefit of one capability decreases as the level of the other capability increases (King et al., 2008; Moorman & Slotegraaf, 1999). From a managerial perspective, the ultimate goal of modern marketing is to create complementarities between DMCs and CMCs to take full advantage of a firm’s capability portfolio (Armstrong et al., 2020). However, DMCs and CMCs can function as complements or substitutes, as the head of digital technologies from a consumer goods company noted:

It depends on how you do it [chuckles]. If I do it badly, digital and classic channels are in opposite directions to each other, they cannibalize each other, and I try to save in one channel what I lost in the other. Ideally, they complement each other, of course, because I also try to develop a holistic view of the customer’s decision-making, purchasing, and usage process, and then control and support this accordingly via classic and digital channels.… The most important thing is to really have a holistic view on the customer.

### A contingency perspective on marketing capabilities

We adopt the contingent view of RBT (e.g., Aragón-Correa & Sharma, 2003) to select relevant contingencies that may either enable or prevent firms from realizing the full potential of their capability portfolio (see Fig. 1). We further support this selection with managerial perspectives from our in-depth interviews (see Table 3). Following Wade and Hulland (2004), we argue that the effective deployment of DMCs and CMCs is dependent on (1) organizational contingencies and (2) environmental contingencies.

**Table 3:** Supportive quotes from in-depth interviews for the selection of contingency factors

Contingency factor	Supportive quotes from in-depth interviews	Interview partner
Customer orientation	Marketing for me clearly means today moving away from what I want to market toward what does the customer need. So a very clear shift completely to the customer’s point of view, and to align everything according to that, the own logic, the internal processes, the budgets and to proceed only according the customers’ input. That’s a very big shift, how marketing is done today.… At the heart of our business model is to develop even more customer-oriented products that give us a unique market position and thus outperform the competition.	Digital marketing manager, electronics industry
Competitor orientation	A clear risk [of digital transformation] that we face is not to be able to deliver product and service innovations that differentiate us from the competition. There are enough companies out there, small start-ups or digital firms like Amazon that innovate at another scale than us…. It comes down to the simple question of being able to keep up with the competition in the long term. Now, we are still able to defend our competitive advantage, however, there is a high risk that other players will show up and disrupt the market. It does not necessarily have to be one of our main competitors, but it can be a firm from a completely different industry.	Marketing manager, electronics industry
Interfunctional coordination	We work more and more cross-functional. Digital marketing, in particular, works almost exclusively across teams.… I have to talk to channel marketing, for example, which is responsible for marketing our products. Then I have to talk to the brand colleagues, who are ultimately responsible for the visualization and presentation of our advertising materials. Then we talk to our web designers, who are also another department.… Then we talk to the editors who put all the content into the content management system. Then there’s a corporate language unit, which ensures that the language is consistent and that it’s understood. So it feels like we’re just exchanging ideas across departments all day long, and it wouldn’t work without that.	Digital marketing manager, information and communication industry
Environmental dynamism	Nowadays technology is changing so quickly that you can’t really plan for it anymore. If we say today, especially in such a large corporation, that we want to launch a new product, it takes up to six years until we have developed and approved it. This means that what I decided yesterday will probably be complete nonsense by the time it actually reaches the customer because it is completely outdated. So one of the key motivating factors of our digital transformation is to better make use of our marketing processes to achieve faster time-to-market of our products and services.	Director marketing and sales, automotive industry

Regarding organizational contingencies, we consider the three core components of market orientation: customer orientation, competitor orientation, and interfunctional coordination (Narver & Slater, 1990). This choice is for two main reasons. First, we aim to shed more light on the relevance of customer in relation to competitor orientation (Moorman & Day, 2016), given that both orientations may be effective in the digital age. On the one hand, customers are more informed, empowered, and demanding than ever before; on the other hand, firms face fierce competition from lower barriers to market entry, the dramatic rise of start-ups and digital firms, and the declining sustainability of competitive advantage (Vial, 2019). To cope with these challenges, managers may put a premium on a customer or competitor orientation, thereby affecting a firm’s ability to leverage its capability portfolio. Second, a recent survey shows that digital marketing practices (e.g., social media marketing, mobile marketing) are led by the marketing function in the majority of firms, whereas for classic marketing practices (e.g., new product development, pricing, sales management), this is the case only in a minority of firms (The CMO Survey, 2019).^3^ Consequently, firms need to interfunctionally coordinate their resources between business functions responsible for DMCs and CMCs to create superior value from their capability portfolio in the marketplace.

Regarding environmental contingencies, we consider the dynamism in a firm’s market environment. We chose environmental dynamism because in rapidly changing environments in the digital age, a comprehensive capability portfolio comprising digital and classic marketing practices may be critical in achieving superior performance (Day, 2011).

## Hypotheses development

### Marketing capabilities and firm performance: Direct effects

#### Effect of DMCs on firm performance

In line with RBT, we argue that DMCs increase firm performance. This is because they are (1) valuable, (2) rare, (3) imperfectly imitable, and (4) organizationally exploitable (Kozlenkova et al., 2014).

First, we argue that DMCs are valuable because they can increase firm profitability beyond what would have been possible without these capabilities (Barney & Hesterly, 2012). Previous empirical research shows that DMCs such as social media marketing or mobile marketing may significantly improve a firm’s revenues and/or cost structure (e.g., Gill et al., 2017; Kumar et al., 2013; Kumar et al., 2016). This improvement occurs because DMCs allow firms to reach, convert, and engage an increasing number of customers by continuously establishing and leveraging interactive linkages in existing and new markets (Wang, 2020). In this way, DMCs steadily provide new revenue-generating opportunities at comparably low deployment costs (Sridhar & Fang, 2019), ultimately increasing firm profitability.

Second, DMCs fulfill the criterion of rarity. For example, survey results among more than 400 marketing executives across industries show not only that DMCs are ranked among the most important marketing capabilities but also that firms have the largest organizational gaps related to their development (The CMO Survey, 2016). Thus, DMCs are in the early stages of development in most firms across industries, and only a few firms possess superior DMCs. Thus, DMCs are rare (Kozlenkova et al., 2014; Wade & Hulland, 2004).

Third, we propose that DMCs meet the condition of imperfect imitability because they foster the social complexity and causal ambiguity of resources. More precisely, DMCs enable firms to generate unique knowledge and relational resources through successive interactions with a high number of customers and partners (i.e., social complexity). As these resources are highly intangible and depend on specific relationships, it is difficult for competitors to untangle the resource combinations responsible for a firm’s success (i.e., causal ambiguity). Kozlenkova et al. (2014) note that social complexity and causal ambiguity are clear indicators that DMCs provide resources that are difficult for competitors to imitate.

Fourth, firms choosing to invest in DMCs typically foster their organizational embeddedness to exploit their potential—that is, the extent to which a capability is contextually entrenched within a firm’s structures and processes (Grewal & Slotegraaf, 2007; Kozlenkova et al., 2014). Specifically, these firms are typically characterized by top management commitment, supporting policies, and performance incentives related to digital marketing (Leeflang et al., 2014). Thus:

#### H1

DMCs are positively related to firm profitability.

#### Effect of CMCs on firm performance

Previous research has repeatedly demonstrated that CMCs are essentially valuable, rare, imperfectly imitable, and exploitable by organizations, thus constituting sources of sustained competitive advantage (Day, 1994; Dutta et al., 1999; Kozlenkova et al., 2014; Krasnikov & Jayachandran, 2008). Empirical studies have also revealed that CMCs make significant contributions to firm profitability (e.g., Dutta et al., 1999; Krasnikov & Jayachandran, 2008; Vorhies et al., 2011). Thus, in line with previous empirical evidence and no further justification, we hypothesize the following:

#### H2

CMCs are positively related to firm profitability.

#### Interaction effect of DMCs and CMCs on firm performance

Previous empirical research shows that digital and classic marketing practices can function as complements (i.e., positive interaction effect) (e.g., Kumar et al., 2017) or substitutes (i.e., negative interaction effect) (e.g., Sridhar et al., 2016), thus improving or reducing firm performance. Next, we elaborate on both perspectives.

On the one hand, DMCs and CMCs can function as complements, which means that, all else being equal, the interaction effect of DMCs and CMCs on firm performance is positive. Prior research indicates that such complementarity is due to better marketing integration resulting from the joint presence of different types of marketing capabilities (e.g., Morgan et al. 2012; Ngo & O'Cass, 2012; Morgan et al., 2009b). Accordingly, we argue that firms with strong DMCs *and* CMCs are better able to foster marketing integration, or the degree to which marketing processes are coordinated across multiple channels and touchpoints to provide customers with a seamless experience (Sousa & Voss, 2006). In practical terms, these firms have better knowledge of the unique benefits and risks associated with the deployment of their DMCs and CMCs in the marketplace, which enables them to better integrate their digital (e.g., social media, mobile, website) and classic (e.g., telephone, store) channels and touchpoints across different stages of the buying process (Lemon & Verhoef, 2016). Such integration likely leads to increased customer loyalty and conversion rates due to more consistent customer interactions, thus improving firm performance (Cao & Li, 2015).

On the other hand, DMCs and CMCs can function as substitutes, which means that, all else being equal, the interaction effect of DMCs and CMCs on firm performance is negative. Prior research implies that such substitutability is due to increased marketing complexity resulting from the joint presence of different types of marketing capabilities (e.g., Eyuboglu et al., 2017; Park & Mithas, 2020). Specifically, firms with strong DMCs *and* CMCs inherently possess a broader capability portfolio that fosters the complexity of marketing processes as determined by the number and configuration possibilities of a firm’s channels and touchpoints (Homburg et al., 2012a; Rivkin, 2001). Such complexity makes it increasingly difficult for managers to identify optimal configurations of digital and classic channels and touchpoints, causing cognitive overload (Eyuboglu et al., 2017; Park & Mithas, 2020). Thus, managers may repeatedly fail to reach customers through their preferred channel and touchpoint configurations. This failure complicates customers’ use of a firm’s channels and touchpoints across different stages of the buying process, thus reducing the number of customer purchases and, ultimately, firm performance (Sridhar et al., 2016; Verhoef et al., 2007). In addition, firms with strong DMCs and CMCs face substantial costs that can outweigh the benefits of their simultaneous deployment, as coordinating a large number of channels and touchpoints requires significant resource investment (Eyuboglu et al., 2017; Valos et al., 2010). Overall, the complexity and costs of managing a broad capability portfolio may be important reasons for substitutive effects between DMCs and CMCs, which can reduce firm performance (Sridhar et al., 2016).

In summary, arguments can be equally made for a positive or negative interaction effect of DMCs and CMCs on firm performance. Therefore, we do not hypothesize this interaction effect. Instead, we propose that organizational and environmental contingencies may strengthen or weaken the interaction effect of DMCs and CMCs beyond a baseline level, thereby differentially affecting firm performance.

### Marketing capabilities and firm performance: Contingency effects

#### Moderating effect of customer orientation

*Customer orientation* refers to the firm’s understanding of its target buyers to create superior value for them on a continuous basis (Narver & Slater, 1990). We argue that customer orientation moderates the interaction between DMCs and CMCs in a positive way.

A customer orientation facilitates a culture of customer data-driven decision-making throughout the firm (Leeflang et al., 2014). Customer-oriented firms continuously capture and combine customer data from DMCs (e.g., social media, mobile apps) and CMCs (e.g., CRM, market research) to generate a unified view of the customer (Kennedy et al., 2003; Neslin et al., 2006). Consequently, customer-oriented firms have a more holistic and in-depth understanding of customers’ needs, behaviors, and buying patterns across different stages of the buying process. Such an understanding fosters a firm’s ability to seamlessly integrate digital and classic channels and touchpoints from the customer’s point of view, which empowers the firm to better reach, convert, and engage with customers at the appropriate point within the buying process. As a result, customer-oriented firms can deploy their DMCs and CMCs more efficiently and effectively in the marketplace, thus driving firm profitability (Järvinen & Karjaluoto, 2015; Neslin et al., 2006).

Furthermore, customer-oriented firms strongly rely on the systematic measurement of customer-based performance metrics, such as customer satisfaction, loyalty, or engagement, across channels and touchpoints (Day & Nedungadi, 1994; Shah et al., 2006). This facilitates the attribution of marketing causes and effects, making it easier for managers to resolve the complexity of the firm’s capability portfolio and identify the optimal configurations of DMCs and CMCs. Therefore, customer-oriented firms are more likely to repeatedly reach their customers through their preferred channel and touchpoint configurations, leading to stronger and more profitable customer relationships. In addition, more profound knowledge of customer-based channel and touchpoint performance enables firms to eliminate channels and touchpoints that provide little value for customers. This reduces the complexity of a firm’s capability portfolio and lowers its management costs (Eyuboglu et al., 2017), ultimately improving its profitability. Thus:

#### H3

Under high levels of customer orientation, the interaction effect of DMCs and CMCs on firm profitability is positive.

#### Moderating effect of competitor orientation

*Competitor orientation* refers to the firm’s understanding of the short-term strengths and weaknesses and long-term capabilities and strategies of its current and potential competitors (Narver & Slater, 1990). Marketing scholars have emphasized that competitor-oriented firms essentially rely on competitive differentiation and competitive imitation to achieve competitive advantage (e.g., Chen & Venkatesh, 2013; Lukas & Ferrell, 2000; Zhou et al., 2009). We argue that competitor orientation may moderate the interaction between DMCs and CMCs in a positive or negative way. Accordingly, we develop two competing hypotheses.

On the positive side, competitor-oriented firms possess substantial knowledge of competitors’ capability portfolios (Day & Nedungadi, 1994), which enables them to better assess the relative strengths and weaknesses of their own configurations of DMCs and CMCs in the marketplace. Therefore, competitor-oriented firms are empowered to expand capability advantages and close capability gaps relative to competitors. This results in a distinctive capability portfolio that provides competitive differentiation (Luo et al., 2007). Equipped with a distinctive capability portfolio and profound knowledge of the relative value of their own configurations of DMCs and CMCs in the marketplace, competitor-oriented firms are able to integrate their digital and classic channels and touchpoints better than competitors. Thus, competitor-oriented firms can provide customers with more unique and differentiated experiences across different stages of the buying process, ultimately increasing firm profitability.

In addition, a competitor orientation may enable firms to imitate configurations of digital and classic channels and touchpoints that have proved profitable in the marketplace (Johnson & Bharadwaj, 2005). Such competitive imitation provides firms with a convenient source of marketing best practices and significantly reduces the substantial costs of trial-and-error learning (Lukas & Ferrell, 2000). Thus, a competitor orientation may enable firms to reduce the complexity of a firm’s capability portfolio by launching proven configurations of DMCs and CMCs and avoiding the costly implementation and elimination of unprofitable channel and touchpoint configurations. In this way, competitor orientation enhances the efficiency and effectiveness of a firm’s capability portfolio, thus improving firm profitability.

On the negative side, imitation of competitors’ channel and touchpoint configurations may have a detrimental impact on firm profitability. According to RBT, the social complexity and causal ambiguity of market-based resources make it difficult to identify the right configurations of DMCs and CMCs that are responsible for another firm’s success in the marketplace (Kozlenkova et al., 2014; Rivkin, 2000). Adding to the difficulty, managers generally seek to imitate best-in-class firms (Ordanini et al., 2008)—such as pure players or start-ups—that combine unique skills, knowledge, and organizational routines across various departments to create superior customer experiences.^4^ Therefore, firms are likely to fail to identify and implement the right capability configurations, leaving competitors’ successful combinations unmatched (Rivkin, 2000). However, “the cost of combining resources in the wrong way is particularly high” (Ordanini et al., 2008, p. 387) because the adopted capability configurations may not be valuable, such that their implementation and maintenance costs exceed their revenue contributions, thereby reducing firm profitability. Even if the right configurations of DMCs and CMCs can be identified, incumbent firms often face implementation difficulties due to legacy IT-systems, functional silos, or cultural barriers (Vial, 2019). Poor implementation of channel and touchpoint configurations at the customer interface, however, may result in coordination difficulties of customer-facing processes in different stages of the purchase journey. This leads to less integrated and thus fragmented customer interactions, which may decrease customer loyalty or lower conversion rates, ultimately reducing profitability (Cao & Li, 2015; Verhoef et al., 2007).

Aside from reducing a firm’s marketing integration, a competitor orientation may increase the complexity of a firm’s capability portfolio. Competitor-oriented firms view the marketplace as a battlefield and strive to defeat competitors, sometimes even at the expense of their own profits (Armstrong & Collopy, 1996; Luo et al., 2007). Consistent with this line of reasoning, Geyskens et al. (2002, p. 102) note that “marketing channels are powerful weapons in an increasingly competitive battle for consumers. An important way in which companies use these weapons is by adding new channels to existing ones.” Consequently, competitor-oriented firms are more likely to add new or preserve existing configurations of digital and classic channels and touchpoints to beat the competition. This is because a firm’s channels and touchpoints are key facets of competitive differentiation and can provide sustainable competitive advantage (Homburg et al., 2014; Lemon & Verhoef, 2016). Additional channels and touchpoints, however, increase complexity and make it even more difficult for managers to identify the optimal configurations of a firm’s DMCs and CMCs (Sousa & Voss, 2006). Therefore, managers are less likely to reach customers through their preferred channel and touchpoint configurations. As a result, a competitor orientation may decrease the efficiency and effectiveness of a firm’s capability portfolio, thus reducing firm profitability. Given these competing viewpoints, we formulate the following:

#### H4a

Under high levels of competitor orientation, the interaction effect of DMCs and CMCs on firm profitability is positive.

#### H4b

Under high levels of competitor orientation, the interaction effect of DMCs and CMCs on firm profitability is negative.

#### Moderating effect of interfunctional coordination

*Interfunctional coordination* refers to the coordinated use of firm resources in creating superior value for target customers (Narver & Slater, 1990). Interfunctional coordination essentially fosters the integration of resources toward a unified understanding of how to create superior customer value and the communication of successful and unsuccessful customer experiences across business functions (Narver & Slater, 1990). We argue that interfunctional coordination moderates the interaction between DMCs and CMCs in a positive way.

Interfunctional coordination enables firms to continuously integrate customer-related knowledge and relational resources between DMCs and CMCs that are typically dispersed throughout the firm (Homburg et al., 2015; The CMO Survey, 2018). In this way, interfunctional coordination helps managers oversee all customer interactions across business functions (e.g., marketing, sales, customer service). A holistic understanding of customer interactions increases the consistency of information exchanged and transactions conducted with the customer across different digital and classic channels and touchpoints (Jayachandran et al., 2005; Sousa & Voss, 2006). As a result, firms can provide customers with more seamless experiences at different stages of the buying process (Lemon & Verhoef, 2016), ultimately increasing firm profitability.

Moreover, interfunctional coordination helps firms reduce the complexity of their capability portfolios. This is because sharing of successful and unsuccessful customer experiences across all business functions (Narver & Slater, 1990) (e.g., marketing, sales, customer service) facilitates managers’ holistic understanding of the benefits and risks associated with their firms’ digital and classic channels and touchpoints. Therefore, managers are better able to evaluate which individual channels and touchpoints to use and in what configurations to create superior value for different customer segments at different stages of the buying process (Payne & Frow, 2004). Interfunctional coordination thus reduces complexity by facilitating the identification of optimal configurations of a firm’s DMCs and CMCs. As a result, managers are more likely to repeatedly reach customers through their preferred channel and touchpoint configurations, ultimately increasing firm profitability.

#### H5

Under high levels of interfunctional coordination, the interaction effect of DMCs and CMCs on firm profitability is positive.

#### Moderating effect of environmental dynamism

*Environmental dynamism* is the rate at which customer preferences, competitor actions, and technologies change in the marketplace (Jaworski & Kohli, 1993). We argue that environmental dynamism moderates the interaction between DMCs and CMCs in a positive way.

In dynamic environments, customers more frequently shift between digital and classic channels and touchpoints across different stages of the buying process, which puts a premium on seamless customer interactions (Verhoef et al., 2021). Thus, channel and touchpoint integration resulting from strong DMCs and CMCs is more critical to satisfy customer needs and achieve superior performance. Consequently, when firms are operating in dynamic environments, the simultaneous deployment of DMCs and CMCs should be more strongly related to firm profitability.

Moreover, a more complex capability portfolio resulting from strong DMCs and CMCs enhances a firm’s ability to successfully cope with dynamic environments (Neslin et al., 2006). This is because firms with a higher number of channels and touchpoints are more likely to repeatedly provide different customer segments with the channel and touchpoint configurations that optimally satisfy their changing preferences at different stages of the buying process (van Bruggen et al., 2010). This results in stronger customer relationships, ultimately improving firm profitability. In addition, a higher number of channels and touchpoints resulting from strong DMCs and CMCs fosters a firm’s ability to quickly adapt and respond to changing technologies and competitor actions in the marketplace. Against this background, we propose that in dynamic environments, the benefits of a more complex capability portfolio resulting from strong DMCs and CMCs likely outweigh the associated management costs, thus increasing firm profitability. Consistent with this line of reasoning, Eyuboglu et al. (2017) empirically demonstrate that high channel complexity improves firm profitability in highly dynamic environments. Thus:

#### H6

Under high levels of environmental dynamism, the interaction effect of DMCs and CMCs on firm profitability is positive.

## Research methodology

### Collection of primary and secondary data

We consider the combination of primary manager data and secondary performance data from an objective database as particularly appropriate to test our hypotheses (Morgan et al., 2019). In fact, a key informant approach can provide the most direct measures of marketing capabilities (Danneels, 2016) and has a long tradition in marketing and management research (Conant et al., 1990; Menon et al., 1999; Morgan et al., 2018; Vorhies & Morgan, 2003).

In the first phase, we identified potential respondents for our survey through the online career platform XING. To favor generalizability of our findings, we did not constrain our population of interest to specific industries. Informed by our interviews, we deemed senior and top-level managers with primary responsibility for their firms’ general or digital marketing, sales, customer service, or business activities as competent key informants. We made several efforts to encourage participation and increase response rates (Hulland et al., 2018). To ensure clarity and minimize completion costs, we pretested the design of the survey instrument with four independent academic experts and 18 graduate students with several years of professional experience. In addition, via personalized e-mail we invited potential key informants to participate in our large-scale online survey and followed up with two waves of reminders. Finally, as an incentive we offered informants a study report and a choice between an Amazon.com voucher and a donation to a charity organization. During the five-week response period, we reached 2533 potential respondents and received 382 completed questionnaires (15% response rate). We discarded four observations because key informants failed quality checks.

In the second phase, we collected secondary performance data from Bureau van Dijk’s flagship financial database ORBIS to mitigate common method variance (Podsakoff et al., 2003). We were able to collect independent data for ROA for the majority of firms in our sample (n = 273) to test our hypotheses. Owing to less comprehensive public disclosure requirements, however, we could not obtain financial performance data for several family-, foundation-, or state-owned firms. In the model specification section, we account for a potential selection bias of these sub-samples. Web Appendix B details the sample characteristics.

### Measure development and assessment

#### Marketing capabilities

Following Conant et al. (1990), we carefully designed our marketing capability scales to capture specific marketing activities to clearly differentiate them from the more strategic activities of customer or competitor orientation. According to Morgan (2012), CMCs reflect 11 marketing-mix-related processes, such as product management, pricing management, sales channel management, and marketing communications. Vorhies and Morgan (2005) empirically show that CMCs—captured as the interdependence between marketing-mix-related processes—exert a superior impact on firm performance. This finding is in line with RBT, which states that an interdependent set of marketing capabilities can provide unique value to the firm (e.g., Amit & Schoemaker, 1993; Srivastava et al., 1999). Under this theoretical lens, CMCs are understood as “a second-order construct capturing the covariance among” (Vorhies & Morgan, 2005, p. 83) marketing-mix-related processes. Such treatment of CMCs as a multidimensional construct has a long tradition in marketing capability research (e.g., Acikdilli et al., 2020; Martin & Javalgi, 2019; Martin et al., 2020; Morgan et al., 2004; Morgan et al. 2012; Vorhies et al., 2011). Our study follows this tradition. Specifically, we conceptualize and operationalize CMCs as a second-order construct reflected by the three first-order dimensions of product/service management and development, pricing and sales management, and market research and communication management.^5^

Given the sparseness of empirical research on digital marketing, we could not draw on existing scales, so we developed a new measure for DMCs. We followed established scale development procedures as applied in previous marketing and management research on marketing capabilities (e.g., Conant et al., 1990; Krush et al., 2015; Vorhies et al., 2011; Vorhies & Morgan, 2003). As enumerating all capabilities is not possible, “because every business develops its own configuration of capabilities that is rooted in the realities of its competitive market, past commitments, and anticipated requirements” (Day, 1994, p. 40), we aimed to identify digital core marketing capabilities commonly used and recognized by practitioners across industries. First, we conducted a thorough literature review of academic and business literature to develop a preliminary definition of our focal construct. Second, we used managerial insights from our 49 in-depth interviews to gain a deeper understanding of digital core marketing capabilities. Unifying our literature-based and managerial insights, we carefully developed a comprehensive set of items that capture the focal construct’s domain. To assess content validity, we presented the items to three independent academic experts and 12 executive MBA students and made changes in line with their feedback. We model DMCs as a one-dimensional reflective construct^6^ that captures seven digital core marketing capabilities: social media marketing, mobile marketing, content marketing, search engine marketing, web analytics, marketing automation, and e-mail marketing. These capabilities were consistently confirmed by the existing literature, our in-depth interviews, and results of pretesting with academics and managers. Consistent with prior research (e.g., Vorhies et al., 2011; Vorhies & Morgan, 2003), we assessed CMCs and DMCs relative to competitors.

#### Moderating variables

We measured customer orientation, competitor orientation, and interfunctional coordination each with items adapted from Narver and Slater (1990). For environmental dynamism, we used items from Jaworski and Kohli (1993).

#### Firm performance

To measure firm profitability we used ROA, which is the ratio of profit to total assets. Specifically, we used industry-adjusted ROA by subtracting the industry mean ROA from the individual firm ROA (Vorhies et al., 2011). We captured this variable one year after the survey (t + 1).

#### Control variables

We include control variables that have frequently been used in previous marketing capability studies at both the firm and industry level (Kamboj & Rahman, 2015). At the firm level, we control for the effect of firm size and firm age, as larger firms may have more slack resources and older firms may have more knowledge and relational resources (e.g., Vorhies et al., 2011). In addition, we control for firm type (i.e., B2B vs. B2C) to account for the multifaceted differences between B2B and B2C contexts (Lilien, 2016). Furthermore, we control for structural flux to capture a firm’s transformation activities in terms of the rate at which an organization changes its structure, rules, personnel, and procedures (Maltz & Kohli, 1996). At the industry level, we control for whether firms operate in product- or service-oriented industries to consider the unique characteristics between these settings (Zeithaml et al., 1985). We also control for whether industries are strongly or weakly influenced by digital business transformation to account for differences in terms of pressure to offer digital products or services (Edeling & Himme, 2018).

#### Measure assessment

To determine the reliability and validity of our measures, we conducted confirmatory factor analyses (CFAs). First, we conducted a separate CFA (e.g., Vorhies et al., 2011) for the second-order construct CMCs. Second, after validating this construct, we used item parceling and averaged the items on the level of each first-order dimension to develop an aggregated scale (Bagozzi & Edwards, 1998). Third, we performed one CFA on all multi-item constructs and found satisfactory model fit: CFI = .92; TLI = .91; RMSEA = .05. Overall, all standardized factor loadings were high and significant (*p* < .01), and all Cronbach’s alphas (CA) and composite reliabilities (CR) were above the required thresholds, thus providing evidence for convergent validity. Discriminant validity is demonstrated, as the square root of the average variance extracted (AVE) of each construct exceeds the correlation between itself and any other construct (Fornell & Larcker, 1981). In this way, we demonstrate that DMCs and CMCs are empirically distinct constructs. Table 4 shows means, standard deviations, and measurement properties of our measures, and Table 5 reports correlations^7^ and the square roots of the AVEs. Web Appendix C details the operationalization of our measures.

**Table 4:** Descriptive statistics

Variables	M	SD	CA	CR
Multi-item measures				
Digital marketing capabilities	3.03	.67	.86	.86
Classic marketing capabilities	3.25	.57	.76	.76
Customer orientation	4.05	.80	.89	.89
Competitor orientation	3.54	.81	.78	.79
Interfunctional coordination	3.47	.81	.80	.81
Environmental dynamism	3.46	.89	.88	.88
Structural flux	2.65	.88	.86	.86
Objective performance measures				
ROA (t + 1) [%]	6.78	8.68		
Other measures				
Firm size [log]	9.53	2.48		
Firm age [log]	3.67	1.00		
B2B vs. B2C [%]	71.85	35.66		
Product vs. service [%]	67.52	36.15		
Relevance of digital business transformation	.31	.46		

Notes: CA = Cronbach’s alpha; CR = composite reliability. The values are based on our full sample of 378 firms. The values for ROA (t + 1) and firm size are based on 273 firms. The values for the variables firm size and firm age are logarithmically transformed. The variables ROA (t + 1) and firm size are retrieved from the ORBIS database

**Table 5:** Correlations

Variables	1	2	3	4	5	6	7	8	9	10	11	12	13
1. Digital marketing capabilities	*.69*												
2. Classic marketing capabilities	.65^***^	*.72*											
3. Customer orientation	.32^***^	.51^***^	*.82*										
4. Competitor orientation	.39^***^	.51^***^	.57^***^	*.70*									
5. Interfunctional coordination	.29^***^	.46^***^	.54^***^	.60^***^	*.71*								
6. Environmental dynamism	.16^***^	.11^**^	.13^**^	.21^***^	.19^***^	*.74*							
7. Structural flux	.08	.00	−.05	.01	.03	.30^***^	*.71*						
8. Firm size	.06	.10^*^	.05	.06	.04	.05	.13^**^	–					
9. Firm age	.00	.01	.00	.00	−.05	−.11^**^	−.16^***^	−.07	–				
10. B2B vs. B2C	−.07	−.08	.00	−.05	.04	−.25^***^	−.07	−.10^*^	.05	–			
11. Product vs. service	.03	.03	−.04	.01	.01	−.15^***^	−.12^**^	−.05	.05	.03	–		
12. Relevance of digital business transformation	.06	−.02	.08	.04	.03	.30^***^	.12^**^	−.04	−.10^**^	−.04	−.26^***^	–	
13. ROA (t + 1)	.23^***^	.21^***^	.07	.05	.06	−.02	.07	−.05	.01	.01	.05	−.06	–

^*^p < .10, ^**^p < .05, ^***^p < .01

Notes: We display the square root of the average variance extracted on the diagonal, if applicable. Correlations are based on our full sample of 378 firms. The correlations for ROA (t + 1) and firm size are based on 273 firms. The variables ROA (t + 1) and firm size are retrieved from the ORBIS database

## Empirical results

### Model specification

We employ regression analyses to test our hypotheses. Details of the model specification appear in Web Appendix D. Before testing the hypothesized relationships, we checked for non-response bias, key informant bias, and key potential sources of endogeneity. Overall, we have strong indications that these threats are unlikely to bias the results of our study.

#### Non-response bias

For all multi-item constructs, Armstrong and Overton’s (1977) test yielded no significant differences between early and late respondents. Similarly, results show no significant differences in terms of industry between the responding firms and the firms we initially addressed. This indicates that non-response bias is not an issue in our data.

#### Key informant bias

To strengthen confidence in key informant quality, we followed four approaches. First, we made sure to target respondents with a relatively high hierarchical position. Most key informants (71.2%) had titles of head of department or higher (see Web Appendix B). Second, we checked for key informant competency by asking about job experience. Respondents’ average job experience in their firm was 9.8 years and 5.2 years in their current position, suggesting that informants were well experienced. In addition, we directly asked informants about their competence in answering all the survey questions. The results suggest that 70% of key informants were highly competent and another 26% were competent, indicating that informants were well qualified overall to report on the survey.^8^ As key informant accuracy increases with hierarchical position and tenure (Homburg et al., 2012c), these results suggest that the informants are knowledgeable about their firms’ capabilities and other study variables. Third, we ensured that key informants understood and could accurately answer the survey questions by carefully pretesting the questionnaire. Fourth, we verified informants’ responses by comparing primary manager and secondary data on sales revenue. The high correlation coefficient (r = .70, *p* < .001) shows that key informant bias is not a problem.

#### Endogeneity

We accounted for several potential sources of endogeneity. First, our research design greatly reduces the risk of potential common method variance (CMV), as we relied on survey data to capture firm capabilities and secondary data for firm performance (Podsakoff et al., 2003; Rindfleisch et al., 2008). Importantly, with respect to the investigated interaction effects, research has shown that “interaction effects cannot be artifacts of CMV” (Siemsen et al., 2010, p. 456) because CMV “reduces the power to find significant interactions effects” (Palmatier, 2016, p. 656). In addition, we applied several procedural remedies to alleviate potential CMV. Specifically, we carefully pretested the questionnaire, separated the independent and moderating variables, and assured respondents of the anonymity and confidentiality of their responses (Hulland et al., 2018; Podsakoff et al., 2003). Second, to account for potential sample-induced endogeneity, we applied the Heckman (1979) selection procedure with respect to our empirical model. Following Homburg et al. (2012b), we used the legal form of the company because it reflects disclosure requirements for identification and the availability of secondary data as the dependent variable. We obtained the inverse Mills ratio (*λ*), which we include in the analyses. Third, to prevent reverse-causality concerns, we established a clear temporal order between our variables. Following conceptual considerations based on the marketing performance–outcomes chain (Katsikeas et al., 2016), we used a one-year time lag to assess the effects of marketing capabilities (t) on firm profitability (t + 1).

### Hypotheses testing

In Table 6, we report the results of the hierarchical ROA model estimation process, which includes control variables (Model 1), main effects (Models 2 and 3), and three-way interactions (Model 4). We mean-centered all variables before creating the interaction terms. We also checked for multicollinearity, which does not seem to threaten the validity of our results because variance inflation factors for the regression models are below the commonly accepted threshold value of 10 (Hair et al., 2010).

**Table 6:** Regression results

Dependent variable	ROA (t + 1)				
	Model 1	Model 2	Model 3	Model 4	
Main effects					
Digital marketing capabilities (DMCs)			.19^**^	.27^***^	H1 (✓)
Classic marketing capabilities (CMCs)		.27^***^	.16^**^	.16^**^	H2 (✓)
Three-way interaction effects					
DMCs × CMCs × CUST				.35^**^	H3 (✓)
DMCs × CMCs × COMP				−.44^***^	H4b (✓)
DMCs × CMCs × IFC				−.06	H5 (−)
DMCs × CMCs × ED				.16^**^	H6 (✓)
Control effects					
Two-way interaction effects					
DMCs × CMCs				−.03	
DMCs × CUST				−.01	
DMCs × COMP				.32^**^	
DMCs × IFC				−.16	
DMCs × ED				.07	
CMCs × CUST				.20^*^	
CMCs × COMP				−.23^*^	
CMCs × IFC				.05	
CMCs × ED				−.01	
Controls					
Customer orientation (CUST)	.06	−.02	−.02	−.01	
Competitor orientation (COMP)	−.01	−.09	−.12	−.04	
Interfunctional coordination (IFC)	.03	.01	.04	.00	
Environmental dynamism (ED)	−.04	−.04	−.05	−.09	
Structural flux	.08	.07	.07	.04	
Firm size	−.14	−.16	−.12	−.09	
Firm age	.04	.04	.03	.00	
Product vs. service	.00	.00	.00	.01	
B2B vs. B2C	−.03	−.02	.00	.03	
Relevance of digital business transformation	−.07	−.07	−.08	−.10	
Endogeneity correction					
Inverse Mills ratio	−.10	−.11	−.06	−.05	
R^2^	.03	.07	.09	.19	
∆R^2^		.04	.02	.10	
F-change		12.87^***^	5.08^**^	2.28^***^	

^*^p < .10, ^**^p < .05, ^***^p < .01

Notes: Standardized coefficients are shown. H (✓) = hypothesis supported, H (−) = hypothesis not supported. The dependent variable ROA (t + 1) is industry-adjusted. The results are based on the sub-sample of 273 firms

The results of Model 3 reveal a positive relationship between DMCs and ROA (b = .19, *p* < .05), in support of H1. Similarly, H2 is confirmed (b = .16, *p* < .05), as the main effect of CMCs on ROA is statistically significant. As Table 6 indicates, the increase in R-square from Model 2 to Model 3 due to adding DMCs is statistically significant (ΔR^2^ = .02, *p* < .05), which shows that DMCs contribute to firm performance beyond the influence of CMCs. Though not hypothesized, Model 4 shows that the two-way interaction effect of DMCs and CMCs on firm performance is non-significant (b = −.03, *p* > .10). As indicated by the positive three-way interaction in Model 4, customer orientation positively moderates the interaction effect of DMCs and CMCs (b = .35, *p* < .05), in support of H3. However, the opposite is true for competitor orientation, as indicated by the negative three-way interaction among DMCs, CMCs, and competitor orientation in Model 4, in support of H4b (b = −.44, *p* < .01). As such, H4a is not supported. Similarly, H5 is not supported, as the three-way interaction among DMCs, CMCs, and interfunctional coordination in Model 4 is not significant (b = −.06, *p* > .10). Finally, Model 4 reveals that the three-way interaction among DMCs, CMCs, and environmental dynamism is significantly positive (b = .16, *p <* .05), in support of H6.

**Fig. 2:**
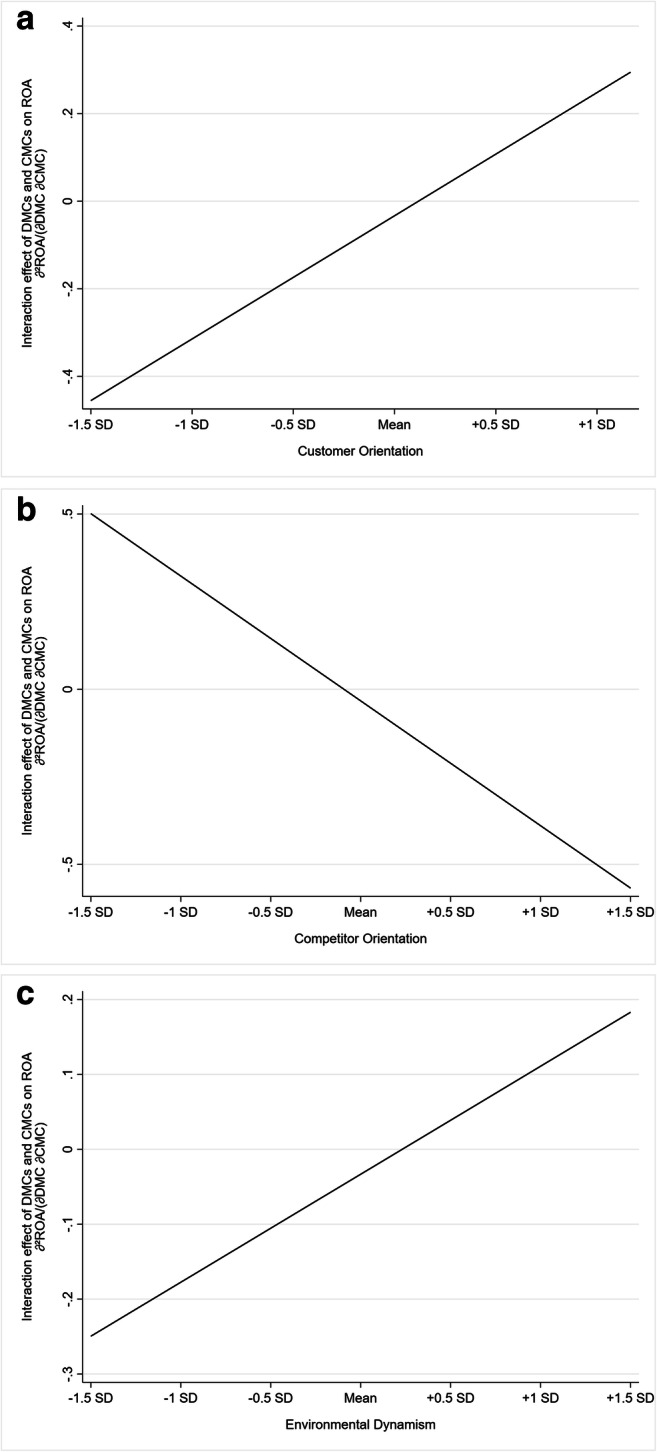
Three-way interaction effects. **Panel A:** Interaction effect of DMCs and CMCs on ROA over the range of customer orientation (H3). **Panel B:** Interaction effect of DMCs and CMCs on ROA over the range of competitor orientation (H4). **Panel C:** Interaction effect of DMCs and CMCs on ROA over the range of environmental dynamism (H6)

To illustrate the significant three-way interaction effects, we follow Schoonhoven’s (1981) partial derivative approach previously applied in marketing research (e.g., Noordhoff et al., 2011; Wathne & Heide, 2004) and evaluate the interaction effect of DMCs and CMCs across the range of values of the moderators. Figure 2 depicts the respective relationships. As Panel A indicates, the interaction effect of DMCs and CMCs is significantly negative at low values (b_–1.5 SD_ = −.45, *p* < .05; b_–1 SD_ = −.31, *p* < .10) and significantly positive at high values (b_1 SD_ = .24, *p* < .05; b_max_ = .29, *p* < .05) of customer orientation.^9^ Panel B indicates that the interaction effect of DMCs and CMCs on ROA is significantly positive at low values (b_–1.5 SD_ = .50, *p* < .05; b_–1 SD_ = .32, *p* < .05) and significantly negative at high values (b_1 SD_ = −.39, *p* < .05; b_1.5 SD_ = −.57, *p* < .01) of competitor orientation. Finally, Panel C shows that the interaction effect of DMCs and CMCs is negative at low values (b_–1.5 SD_ = −.25, *p* < .05; b_–1 SD_ = −.17, *p* > .10) and significantly positive at high values (b_1 SD_ = .11, *p* < .10; b_1.5 SD_ = .18, *p* < .05) of environmental dynamism.

### Post hoc analyses

#### Robustness checks

To further ascertain the robustness of our findings, we reestimated our regression models using alternative accounting-based performance and market-based performance measures. Regarding accounting-based performance measures, we chose return on capital employed (ROCE) because it “[taps] the same theoretical domain” (Richard et al., 2009, p. 739) as ROA and therefore is well-suited for a robustness check. Furthermore, we rely on return on equity (ROE) because it represents returns to stockholders and therefore is “the true bottom-line measure of firm performance” (Ross et al., 2001, p. 59). In addition, we chose return on sales (ROS) because it is a popular accounting-based performance measure in marketing research (Katsikeas et al., 2016). Regarding market-based performance measures, we rely on Tobin’s q^10^ for all publicly traded firms in our sample (n = 112) because it reflects a firm’s long-term performance (Katsikeas et al., 2016) and “is risk adjusted, independent of industry, and provides a good indicator of shareholder value” (Morgan and Rego 2006, p. 427). For ROCE, ROE, and ROS all hypothesized effects identified in the main model remain robust. In contrast, for Tobin’s q, four out of six effects remain robust, with H2 and H6 not supported. This may be because marketing capabilities affect more directly accounting-based performance measures than market-based performance measures (Katsikeas et al., 2016). The reason for this is that accounting-based performance measures are more closely related to the internal functioning of a firm, whereas market-based performance measures rely on external investors’ preferences and expectations of a firm’s value (Bamberger et al., 2021). Taken together, our results are largely robust to different firm profitability measures, providing additional empirical support for our findings (see Web Appendix E).

#### Test of the moderating effect of firm type

Considering the multifaceted differences between B2B and B2C contexts (Lilien, 2016), we investigate whether the interaction effect of DMCs and CMCs on firm profitability varies across these settings. Thus, we reestimated our ROA model and included the three-way interaction among DMCs, CMCs, and firm type. The results reveal that the two-way interaction effects between DMCs and firm type and CMCs and firm type are both non-significant (*p* > .10). In contrast, the respective three-way interaction effect is significantly negative (b = −.19, *p* < .01), thus indicating that B2B firms generate lower payoffs from the deployment of DMCs *and* CMCs than B2C firms.

## Discussion

### Research contributions and implications

Although scholars and practitioners have repeatedly stressed the importance of DMCs for firms to remain competitive in increasingly digital market environments, these capabilities have so far received inadequate research attention. By drawing on a mixed-methods approach, we contribute to and extend prior marketing research in three important ways.

First, we provide a new perspective on the differences between DMCs and CMCs by synthesizing literature- and interview-based insights. Our qualitative inquiry reveals that DMCs and CMCs differ in terms of (1) scalability, (2) measurability, (3) interconnectivity, and (4) adaptability (see Table 2). In this way, we provide a conceptual distinction between DMCs and CMCs and extend existing knowledge on marketing capabilities.

Second, our study broadens and deepens the understanding of whether DMCs contribute to firm performance beyond the effects of CMCs. In contrast with most previous studies (see Table 1), we considered both constructs and conceptualized them across a broad set of marketing activities to foster the generalizability of our results. By drawing on a multi-industry, multisource dataset collected at two points in time, we provide strong empirical support for the value relevance of DMCs to firm profitability. Thus, we add to the marketing literature by contributing to Verhoef and Bijmolt’s (2019) call for research to identify the key capabilities that drive firm performance in the digital age.

Third, our study represents a pioneering empirical effort by developing a contingency perspective on the interaction between DMCs and CMCs. Prior studies in the advertising context reveal that digital and classic marketing practices can have a non-significant (e.g., Bayer et al., 2020), positive (e.g., Kumar et al., 2017), or negative (e.g., Sridhar et al., 2016) interaction effect on firm performance. Given these conflicting empirical results, we theoretically argue that DMCs and CMCs can jointly benefit or hurt firm profitability, depending on moderating contingencies. Empirically, we show that the two-way interaction effect of DMCs and CMCs on firm performance is non-significant (see Table 6, Model 4). The results further reveal that this interaction effect may become positively or negatively significant under certain organizational and environmental contingencies, underscoring the importance of considering three-way interactions when investigating capability complementarity and substitution effects.

Regarding organizational contingencies, this study reveals that customer orientation is critical to unlock the contingent value of DMCs and CMCs, as their simultaneous deployment has a positive influence on firm profitability when customer orientation is high. By contrast, our additional analyses in the hypotheses testing section reveal that the simultaneous deployment of DMCs and CMCs has a negative impact on firm profitability when customer orientation is low (see also Fig. 2, Panel A). Previous research suggests that firms must not only possess distinctive capabilities to achieve superior performance but also a matching organizational orientation to leverage those capabilities (e.g., Newbert, 2007; Wiklund & Shepherd, 2003). In fact, a firm’s organizational orientation reflects its “guiding principles that influence a firm’s marketing and strategy-making activities” (Noble et al., 2002, p. 25)—such as the deployment of marketing capabilities (Menon et al., 1999). Following this line of reasoning, we assume that firms with a low customer orientation may be ineffective in utilizing their marketing capability portfolios in a profitable manner. For instance, these firms may not have incorporated the underlying systems, processes, or metrics to create an in-depth understanding of customers’ needs, behaviors, and buying patterns (e.g., Jayachandran et al., 2005; Morgan et al., 2005; Shah et al., 2006). Lacking this in-depth customer understanding, however, firms are ineffective in identifying the combinations of digital and classic channels and touchpoints that match the preferences and behaviors of distinct customer segments at different stages of the buying process (Lemon & Verhoef, 2016). As a result, firms with low customer orientation may repeatedly fail to reach customers through their preferred channel and touchpoint configurations, thus weakening customer relationships and ultimately firm profitability. In this way, firms’ marketing capability portfolios remain underutilized, such that their development and maintenance costs exceed their revenue contributions.

Furthermore, we find that competitor orientation hampers the deployment of DMCs and CMCs, resulting in lower levels of firm profitability (see also Fig. 2, Panel B). This finding is consistent with previous empirical studies confirming a negative impact of competitor orientation on firm profitability (e.g., Armstrong & Collopy, 1996). The empirical analyses thus argue against the positive side and in favor of the negative side of competitor orientation in deploying DMCs and CMCs. This is presumably because (1) competitive imitation of capability configurations is likely to fail due to the social complexity and causal ambiguity of market-based resources and (2) competitive differentiation gives firms a false incentive to increase complexity by adding new or preserving existing configurations of DMCs and CMCs to beat the competition.

Recently, Moorman and Day (2016, p. 25) raised the question whether the increasing emphasis on customer orientation and de-emphasis on competitor orientation in the digital age “endanger the performance effects of market-oriented culture” or “unleash an even stronger performance effect.” By identifying divergent moderating effects of customer orientation (i.e., positive effect) and competitor orientation (i.e., negative effect) on the interaction between DMCs and CMCs, we contribute to answering this research question.

Moreover, we find no support for the profitability-enhancing impact of interfunctional coordination on the interaction between DMCs and CMCs. We believe that this may be for two main reasons. First, previous research points out that higher levels of interfunctional coordination may incur significant costs associated with the cross-functional management of resources (Lee et al., 2015). These costs may offset the potential benefits of interfunctional coordination in terms of greater integration and reduced complexity between a firm’s DMCs and CMCs, such that the moderating impact of interfunctional coordination is non-significant. Second, there may be other types of interfunctional coordination through which firms achieve integration between DMCs and CMCs that we did not capture in our study. We used Narver and Slater’s (1990) measure of interfunctional coordination, which captures the value creation emphasis of a firm’s integration activities (Im & Nakata, 2008). Other types of interfunctional coordination may emphasize further integration activities related to the compatibility of information systems, quality of exchanged information, or harmony of relationships between functions responsible for DMCs and CMCs (e.g., Song et al., 2000).

Regarding environmental contingencies, we show that the simultaneous deployment of DMCs and CMCs is particularly valuable when firms face rapid changes in customer preferences, competitor actions, and technologies in their market environment. Specifically, in dynamic environments, the benefits of a broad capability portfolio in terms of increased marketplace differentiation and adaptation appear to outweigh the associated development and maintenance costs, thus increasing firm profitability. In stable environments, however, the simultaneous deployment of DMCs and CMCs hurts firm profitability, as outlined previously in our hypotheses testing section (see also Fig. 2, Panel C). This is likely because under these conditions, firms can achieve sufficient pay offs from a smaller number and less differentiated channel and touchpoint configurations. One important reason for this may be that customers less frequently shift between digital and classic channels and touchpoints across different stages of the buying process, as customer needs tend to be homogeneous and change slowly. Similarly, longer technology life cycles and a relatively stable competitive landscape allow companies with a narrower, less complex capability portfolio to adapt sufficiently quickly to marketplace changes (Eyuboglu et al., 2017). In other words, in stable environments, firms have no need to possess broader, more complex capability portfolios, and therefore the associated development and maintenance costs are unjustified.

In summary, our research confirms the “untested proposition … that the capabilities for making and implementing marketing mix decisions [i.e., CMCs] will become more effective and timely when guided by adaptive marketing capabilities [i.e., DMCs]” (Day, 2011, p. 194). In contrast with this bright side, we show that the simultaneous deployment of DMCs and CMCs also has a dark side, as it may hurt profitability.

### Future research directions and limitations

As with any research, our study is subject to limitations, from which avenues for future research emerge. First, as managers must understand how to build DMCs to achieve higher payoffs, we encourage future research to investigate their antecedents. At the top management level, what is the role of the CMO in driving DMCs, and what are the potential coordination issues with other C-level executives (e.g., chief information officer)? At the employee level, which leadership styles (e.g., transformational vs. transactional) and incentives (e.g., individual vs. team) are most beneficial? When should firms develop DMCs internally and when should they outsource their development to agencies?

Second, additional research should identify positive and negative mediating mechanisms linking DMCs and CMCs with performance (e.g., customer, innovation, firm). Are there different pathways to performance for DMCs and CMCs, and if so, how are these influenced by external and internal contingencies?

Third, further research should extend the set of moderators influencing the interaction between DMCs and CMCs. In particular, we encourage scholars to investigate the role of more stable (e.g., functional, multidivisional structure) and more fluid (e.g., team, network structure) (Lee et al., 2015) organizational structures to provide insights into how different structural types and characteristics affect the deployment of DMCs and CMCs.

Fourth, we relied on a single key informant design and took great care to avoid potential key informant bias. Nevertheless, this research design may pose limitations. Thus, additional research using a secondary key informant design is desirable. Such a design may be particularly helpful in investigating potential thought-world differences or other sources of organizational tension between DMCs and CMCs (Leeflang et al., 2014).

Finally, although our data are not strictly cross-sectional, as we implemented a time lag between the primary and secondary data, future research could extend our findings by drawing on a longitudinal design. A promising avenue for future research is to examine how the performance effects of DMCs and CMCs develop over time and assess related contingencies that may amplify or inhibit their impact on firm performance. In this regard, we encourage scholars to consider the development and deployment costs of DMCs and CMCs and their impact on performance over time (Schilke et al., 2018).

### Managerial implications

Although managers are aware that digital technologies can change a firm’s value creation, roughly two out of three marketing executives still tend to focus on “managing the present” rather than “preparing for the future” (The CMO Survey, 2019). Our empirical findings offer important implications that may advance managerial thinking about DMCs and CMCs.

We clearly demonstrate the financial accountability of DMCs using a cross-industry sample and thus empower managers to justify digital marketing investments to senior management. In particular, the study results reveal that DMCs significantly contribute to accounting-based (e.g., ROA) and market-based (i.e., Tobin’s q) firm profitability metrics—over and above the impact of CMCs. Thus, not only do DMCs help firms more effectively deploy their resources and achieve higher profit levels, but they also seem to be perceived by investors as a strong signal that the firm is shifting to a forward-looking stance.

Moreover, our study shows that the mere presence of strong DMCs *and* CMCs does not result in additional profitability gains that go beyond their individual value contributions. Importantly, we identify certain organizational and environmental contingencies that affect the simultaneous deployment of DMCs and CMCs in a positive or negative way. These results provide important implications for firms pursuing integrated marketing strategies.

Regarding organizational contingencies, managers should be aware that their firms’ orientation constitutes a powerful steering instrument to attain possible joint financial returns from DMCs and CMCs. We find that a customer orientation enables firms to unlock the value-creating potential of DMCs and CMCs while a competitor orientation hampers their interaction effect and ultimately destroys financial value. With these divergent results on the contingent role of customer and competitor orientation, we confirm that “treating the concept of market orientation as an aggregate construct of equally important behavioral orientations can be misleading and limit its strategic value for management practice” (Lukas & Ferrell, 2000, p. 244). Regarding environmental contingencies, managers should recognize that the simultaneous deployment of DMCs and CMCs is particularly profitable for firms operating in rapidly changing environments. By contrast, in stable environments, the costs of maintaining strong DMCs and CMCs across a broad set of marketing activities seem unjustified.

Finally, the results from our post hoc analyses reveal that B2B firms profit less from the simultaneous deployment of DMCs and CMCs than B2C firms. We contend that these performance differences may occur for three reasons (Lilien, 2016): (1) because of the significantly smaller number of potential customers in B2B markets, B2B firms have a lower need for large-scale data analysis in the context of customer segmentation, targeting, and positioning based on DMCs; (2) B2B customers typically expect a significantly higher degree of personal interaction, which puts a premium on personal selling and limits the value potential of deploying DMCs on top of CMCs; and (3) B2B buying processes are highly complex and involve multiple decision-makers, which leads to higher marketing complexity. Another explanation for the performance differences between B2B and B2C firms may be that, in general, B2B firms have less digital marketing knowledge and skills than B2C firms (The CMO Survey, 2021). Furthermore, B2B firms are more likely than B2C firms to suffer from fragmented digital marketing strategies, confusion about ownership of digital roles, and lack of key performance metrics for digital initiatives (Harrison et al., 2017). Consequently, B2B marketing organizations may still be less able than their B2C counterparts to effectively integrate their digital and classic channels and touchpoints and provide customers with seamless experiences. Taken together, although DMCs can be a source of competitive advantage for both B2B and B2C firms—as shown by the positive and significant main effect of DMCs on firm profitability—firms operating in B2C markets may profit more from the simultaneous deployment of DMCs and CMCs.

## Supplementary information

ESM 1 (DOCX 372 KB)

## Footnotes

1 According to Herhausen et al. (2020, p. 276), in the marketing literature, “most attention has been given to the tremendous opportunities digital marketing presents, with little attention on the actual related competences that firms need to be successful.” In a similar vein, a rich body of sales research has been devoted to the adoption, use, and impact of information technology (e.g., customer relationship management, sales force automation) on the sales process (e.g., Ahearne et al., 2007; Ahearne et al., 2008; Park et al., 2010). However, empirical research focusing on recent developments of sales digitalization and digital transformation is scarce, especially in the domain of firm capabilities (Singh et al., 2019).

2 Following Bharadwaj et al. (2013), we view digital technologies as combinations of information, computing, communication, and connectivity technologies.

3 The CMO Survey (2019) conducted among 341 top marketers shows that the marketing function leads digital marketing in 85.7% of firms, social media in 77.8% of firms, and marketing analytics in 75.1% of firms. By contrast, the marketing function leads new products in only 31.7% of firms, pricing in only 23.3% of firms, and distribution in only 10.6% of firms.

4 An example of such imitation is Walmart, which is struggling to copy Amazon’s omnichannel capability portfolio (Yohn, 2017).

5 To provide greater confidence in our model specification, we compared our multidimensional measurement model with a one-dimensional measurement model (e.g., Morgan et al., 2004). The multidimensional measurement model captures CMCs as a second-order construct with 11 items across three first-order dimensions, whereas the one-dimensional measurement model captures CMCs as a first-order construct with 11 items. The results of a chi-square difference test reveal that the fit of the one-dimensional measurement model is significantly worse than the hypothesized multidimensional measurement model (Δd.f. = 3, χ^2^_diff_ = 156.22, *p* < .01). Thus, we find strong empirical support for our conceptualization of CMCs as a second-order construct.

6 Following the advice of an anonymous reviewer, we considered a multidimensional measurement model as an alternative to our one-dimensional measurement model for DMCs. The multidimensional measurement model specifies DMCs as a second-order construct, with one dimension capturing the items related to marketing communications (i.e., social media marketing, mobile marketing, content marketing, and e-mail marketing) and another dimension capturing the items related to marketing analytics (i.e., search engine marketing, web analytics, marketing automation). For identification purposes, we followed Oberecker and Diamantopoulos (2011) and constrained the paths from the second-order factor to the two first-order dimensions to be equal. However, a confirmatory factor analysis modeling DMCs as a second-order construct did not converge. This finding likely indicates an inappropriate measurement model (Baumgartner & Homburg, 1996). In contrast, the one-dimensional measurement scale demonstrates satisfactory convergent and discriminant validity, as outlined in the measurement assessment section and Table 4. In addition, we conducted an exploratory factor analysis that revealed one factor with an eigenvalue higher than 1, thus confirming our one-dimensional measurement model for DMCs. Overall, these analyses provide empirical support for the chosen measurement approach.

7 We report a correlation between DMCs and CMCs of r = .65, which is in line with previous marketing research. For example, Morgan et al. (2012) report a correlation of r = .72 between architectural marketing capabilities and specialized marketing capabilities, and Morgan et al. (2009b) report a correlation coefficient of r = .68 between marketing planning capabilities and marketing implementation capabilities.

8 We reestimated our regression models by dropping the remaining 4% of key informants from our sample who did not rate themselves as highly competent or competent in answering all the survey questions. The results remained essentially the same.

9 For customer orientation, we cannot report the interaction effect of DMCs and CMCs at 1.5 SD above the mean, since this value is outside the range of the observed data. Thus, we examine this interaction effect at the maximum value of the 5-point measurement scale.

10 Consistent with prior research (e.g., Kang et al., 2016), we also controlled for the impact of ROA and R&D intensity (i.e., the ratio of R&D expenditures to sales) on Tobin’s q.
