# Supplementary material for: The value relevance of digital marketing capabilities to firm performance
Source: J Acad Mark Sci. 2022 Apr 20;50(4):666–88. doi: 10.1007/s11747-022-00858-7 (PMC9019537; doi:10.1007/s11747-022-00858-7)
Supplement: Supplementary file 1 — (DOCX 372 KB) [file 11747_2022_858_MOESM1_ESM.docx]

# The value relevance of digital marketing capabilities to firm performance

# Web Appendix

**Web Appendix A** Sample characteristics of in-depth interviews.

|  | **Interview data (n = 49)** |
| --- | --- |
| **Industry** | **%** |
| Information and communication | 20.4 |
| Chemicals | 4.1 |
| Mechanical engineering | 10.2 |
| Automotive | 2.0 |
| Financial and insurance services | 10.2 |
| Retailing | 4.1 |
| Electronics | 18.4 |
| Logistics and transportation | 6.1 |
| Consumer goods | 18.4 |
| Utilities | 2.0 |
| Cross‑industry | 4.1 |
| **Position of respondent** | **%** |
| CXO | 16.3 |
| Vice president/director | 30.6 |
| Head of department | 28.6 |
| Senior manager | 18.4 |
| General manager | 6.1 |
| **Function** | **%** |
| Marketing and sales | 67.3 |
| Digital business | 10.2 |
| General management | 8.2 |
| R&D | 6.1 |
| Other | 8.2 |
| Notes: CXO includes titles such as chief executive officer, chief digital officer, chief marketing officer, and chief financial officer. | |

**Qualitative research design**

The semistructured interviews lasted typically between 30 and 60 minutes and began with general questions about the digital transformation of marketing, followed by a series of specific questions about DMCs and related core capabilities and differences and interactions between DMCs and CMCs. The interviews were audiotaped, transcribed verbatim, and analyzed by independent researchers line-by-line following well-established coding procedures (Strauss and Corbin 1998). First, following Gioia et al. (2013), they identified several first-order categories, which they then organized into second-order themes. Second, they aggregated these themes into overarching dimensions.

**Web Appendix B** Sample characteristics

|  | **Primary data  (n = 378)** | **Secondary data ROA (n = 273)** |
| --- | --- | --- |
| **Industry** | **%** | **%** |
| Information and communication | 15.9 | 16.8 |
| Chemicals | 12.7 | 13.9 |
| Mechanical engineering | 10.9 | 11.0 |
| Automotive | 8.7 | 10.6 |
| Financial and insurance services | 8.2 | 5.5 |
| Retailing | 7.4 | 7.0 |
| Electronics | 5.8 | 6.6 |
| Logistics and transportation | 5.8 | 4.4 |
| Healthcare | 5.6 | 5.5 |
| Consumer goods | 4.2 | 4.8 |
| Building and construction | 2.9 | 2.9 |
| Metal processing | 1.9 | 2.6 |
| Other | 10.0 | 8.4 |
| **Position of respondent** | **%** | **%** |
| CXO | 10.1 | 8.4 |
| Vice president/director | 29.4 | 31.9 |
| Head of department | 31.7 | 31.1 |
| Senior manager | 22.0 | 21.2 |
| General manager | 4.7 | 4.8 |
| Other | 2.1 | 2.6 |
| **Functional affiliation of respondent** | **%** | **%** |
| General marketing | 29.1 | 29.0 |
| General sales | 24.3 | 23.4 |
| Digital unit | 13.5 | 13.6 |
| Customer service | 8.4 | 9.2 |
| Business development | 5.6 | 5.1 |
| Digital marketing | 5.0 | 5.5 |
| Information technology | 2.9 | 3.3 |
| Digital sales | 1.9 | 1.8 |
| R&D | 1.9 | 1.8 |
| Other | 7.4 | 7.3 |
| **Number of employees** | **%** | **%** |
| <200 | 28.8 | 27.5 |
| 200–499 | 13.0 | 12.8 |
| 500–2499 | 20.1 | 20.1 |
| 2500–9999 | 19.1 | 18.0 |
| 10,000–29,999 | 9.5 | 11.0 |
| 30,000–50,000 | 5.0 | 6.6 |
| >50,000 | 4.5 | 4.0 |
| Notes: CXO includes titles such as chief executive officer, chief digital officer, and chief marketing officer. | | |

**Web Appendix C** Operationalization of measures

| Study variables |  |  |  |  | FL |
| --- | --- | --- | --- | --- | --- |

| Digital Marketing Capabilities^a^ |  |  |  |  |
| --- | --- | --- | --- | --- |
| Social media marketing |  |  |  | .63 |
| Mobile marketing |  |  |  | .68 |
| Content marketing |  |  |  | .73 |
| Search engine marketing |  |  |  | .75 |
| Web analytics |  |  |  | .72 |
| Marketing automation |  |  |  | .71 |
| E-mail marketing |  |  |  | .56 |
| Classic Marketing Capabilities^a^ (Morgan 2012; Vorhies and Morgan 2003) | | | |  |
| Product/Service Management and Development |  |  |  |  |
| Product management |  |  |  | .65 |
| Service management |  |  |  | .60 |
| New product development |  |  |  | .69 |
| New service development |  |  |  | .78 |
| Pricing and Sales Management |  |  |  |  |
| Pricing management |  |  |  | .50 |
| Sales channel management |  |  |  | .78 |
| Sales force management |  |  |  | .78 |
| Customer relationship management |  |  |  | .60 |
| Market Research and Communication Management |  |  |  |  |
| Market research |  |  |  | .70 |
| Marketing communications |  |  |  | .56 |
| Brand management |  |  |  | .71 |
| Customer Orientation^b^ (Narver and Slater 1990) | | |  |  |
| Our business objectives are driven primarily by customer satisfaction. | | | | .75 |
| We constantly monitor our level of commitment and orientation to serving customers’ needs. | | | | .83 |
| Our strategy for competitive advantage is based on our understanding of customers’ needs. | | | | .84 |
| Our business strategies are driven by our beliefs about how we can create greater value for customers. | | | | .85 |
| Competitor Orientation^b^ (Narver and Slater 1990) | | |  |  |
| Our salespeople regularly share information within our business concerning competitors’ strategies. | | | | .75 |
| We rapidly respond to competitive actions that threaten us. | | | | .78 |
| Top management regularly discusses competitors’ strengths and strategies. | | | | .70 |
| We target customers where we have an opportunity for competitive advantage. | | | | .56 |
| Interfunctional Coordination^b^ (Narver and Slater 1990) | | | |  |
| Our top managers from every function regularly visit our current and prospective customers. | | | | .59 |
| We freely communicate information about our successful and unsuccessful customer experiences across all business functions. | | | | .72 |
| All of our business functions are integrated in serving the needs of our target markets. | | | | .78 |
| All of our managers understand how everyone in our business can contribute to creating customer value. | | | | .75 |
| Environmental Dynamism^b^ (Jaworski and Kohli 1993) | | | |  |
| Customers’ product and service preferences change quickly over time. | | | | .74 |
| Customers tend to look for new products and services all the time. | | | | .70 |
| One hears of new competitors (e.g., start-ups, incumbents) from direct or adjacent  industries almost every day. | | | | .74 |
| The actions of competitors in our major markets are changing quite rapidly. | | | | .86 |
| The technology in our market is changing rapidly. | | | | .81 |
| Technological changes provide big opportunities in our industry. | | | | .58 |
| Structural Flux^b^ (Maltz and Kohli 1996) | | | |  |
| In our SBU/firm… | | | |  |
| …you never know when your job is going to change. | | | | .65 |
| …the way we do things keeps changing. | | | | .71 |
| …you can never tell when you are going to have a new boss around here. | | | | .70 |
| …the only thing you can be sure of is that something is going to change. | | | | .81 |
| …I am always evaluated based on changing criteria. | | | | .67 |
| …it seems like we are always reorganizing. | | | | .72 |
| Control Variables |  |  |  | **FL** |
| Firm Size^c^ |  |  |  |  |
| Natural logarithm of the number of employees within the SBU/firm. | | | |  |
| Firm Age |  |  |  |  |
| Natural logarithm of the age in years of the SBU/firm. | | | |  |
| B2B vs. B2C (Schilke et al. 2009) | | | |  |
| What percentage of the revenues of your business unit stem from transactions with business  customers (vs. consumers)? [0 – 100%] | | | | |
| Product vs. Service (adapted from Schilke et al. 2009) | | | |  |
| What percentage of the revenues of your business unit stem from selling products (vs.   services)? [0 – 100%] | | | | |
| Relevance of digital business transformation (adapted from Edeling and Himme 2018) | | | |  |
| A dummy variable that classifies industries as strongly (1) or weakly (0) influenced by  digital business transformation. | | | |  |
| R&D Intensity^c^ | | | |  |
| The ratio of R&D expenditures to sales revenue. | | | |  |

^a^5-point scale anchored by “much weaker than competitors” and “much stronger than competitors.”

^b^5-point scale anchored by “strongly disagree” and “strongly agree.”

^c^Retrieved from ORBIS database.

Notes: FL = factor loadings (standardized); SBU = strategic business unit.

**Web Appendix D** Model specification

ROA (t + 1) = β_0_ + β_1_ DMCs + β_2_ CMCs + β_3_ CUST + β_4_ COMP + β_5_ IFC + β_6_ ED + β_7_ DMCs × CMCs + β_8_ DMCs × CUST + β_9_ DMCs × COMP + β_10_ DMCs × IFC β_11_ DMCs × ED + β_12_ CMCs × CUST + β_13_ CMCs × COMP + β_14_ CMCs × IFC + β_15_ CMCs × ED + β_16_ DMCs × CMCs × CUST + β_17_ DMCs × CMCs × COMP + β_18_ DMCs × CMCs × IFC + β_19_ DMCs × CMCs × ED + β_20_ $\lambda$ + β_controls_ CONTROLS + ε,

where ROA is industry-adjusted return on assets, DMCs are digital marketing capabilities, CMCs are classic marketing capabilities, CUST is customer orientation, COMP is competitor orientation, IFC is interfunctional coordination, ED is environmental dynamism, $\lambda$ is the inverse Mills ratio,^[[1]](#footnote-1)^ and CONTROLS includes the variables structural flux, firm size, firm age, product versus service, B2B versus B2C, and relevance of digital business transformation.

**Web Appendix E** Robustness checks

| **Dependent variables** | | **ROCE**  **(t + 1)** | **ROE**  **(t + 1)** | **ROS**  **(t + 1)** | **Tobin’s q**  **(t + 1)** |
| --- | --- | --- | --- | --- | --- |
|  | **Main effects** |  |  |  |  |
| **H1** | Digital marketing capabilities | .15^**^ | .16^**^ | .12^*^ | .14^**^ |
| **H2** | Classic marketing capabilities | .16^**^ | .12^*^ | .13^**^ | –.07 |
|  | **Interaction effects** |  |  |  |  |
| **H3** | DMCs × CMCs × CUST | .40^**^ | .20^**^ | .16^*^ | .39^**^ |
| **H4** | DMCs × CMCs × COMP | –.44^***^ | –.20^*^ | –.21^**^ | –.25^**^ |
| **H5** | DMCs × CMCs × IFC | –.03 | .02 | –.05 | .03 |
| **H6** | DMCs × CMCs × ED | .14^**^ | .10^**^ | .12^*^ | .11 |
| ^*^*p* < .10, ^**^*p* < .05, ^***^*p* < .01.  Notes: Standardized coefficients are shown. ROCE = return on capital employed; ROE = return on equity;  ROS = return on sales; CUST = customer orientation; COMP = competitor orientation; IFC = interfunctional coordination; ED = environmental dynamism. | | | | | |

References

Edeling, A., & Himme, A. (2018). When Does Market Share Matter?: New Empirical Generalizations from a Meta-Analysis of the Market Share-Performance Relationship. *Journal of Marketing, 82*(3), 1–24.

Gioia, D. A., Corley, K. G., & Hamilton, A. L. (2013). Seeking Qualitative Rigor in Inductive Research: Notes on the Gioia Methodology. *Organizational Research Methods, 16*(1), 15–31.

Jaworski, B. J., & Kohli, A. K. (1993). Market Orientation: Antecedents and Consequences. *Journal of Marketing, 57*(3), 53–70.

Maltz, E., & Kohli, A. K. (1996). Market Intelligence Dissemination across Functional Boundaries. *Journal of Marketing Research, 33*(1), 47–61.

Morgan, N. A. (2012). Marketing and Business Performance. *Journal of the Academy of Marketing Science, 40*(1), 102–119.

Narver, J. C., & Slater, S. F. (1990). The Effect of a Market Orientation on Business Profitability. *Journal of Marketing, 54*(4), 20–35.

Schilke, O., Reimann, M., & Thomas, J. S. (2009). When Does International Marketing Standardization Matter to Firm Performance? *Journal of International Marketing, 17*(4), 24–46.

Strauss, A. L., & Corbin, J. M. (1998). *Basics of qualitative research: Techniques and procedures for developing grounded theory* (2nd ed.). Thousand Oaks: Sage Publications.

Vorhies, D. W., & Morgan, N. A. (2003). A Configuration Theory Assessment of Marketing Organization Fit with Business Strategy and Its Relationship with Marketing Performance. *Journal of Marketing, 67*(1), 100–115.

1. The inverse Mills ratio is the ratio of the probability density function to the cumulative density function. [↑](#footnote-ref-1)
